# Nutritional Profile and Functional Characteristics of Various Types of Traditionally Produced PEKMEZ—Part I

**DOI:** 10.1002/fsn3.70662

**Published:** 2025-07-16

**Authors:** Nazik Meziyet Dilek, Ali Samet Babaoğlu, Kubra Unal, Metin Turan, Lütfi Pırlak, Mustafa Karakaya

**Affiliations:** ^1^ Department of Nutrition and Dietetics Akşehir Kadir Yallagöz School of Health, Selçuk University Konya Türkiye; ^2^ Department of Food Engineering, Agriculture Faculty Selçuk University Konya Türkiye; ^3^ Department of Agricultural Trade and Management Faculty of Economy and Administrative Sciences, Yeditepe University Istanbul Türkiye; ^4^ Department of Horticulture Agriculture Faculty, Selçuk University Konya Türkiye

**Keywords:** amino acid composition, fruit‐based syrups, traditional products, vitamin content

## Abstract

This study aimed to evaluate the nutritional profile and functional characteristics of eight traditional pekmez varieties produced from different fruits. Samples of andiz, pear, aronia, white mulberry, black mulberry, carob, pomegranate, and grape pekmez were obtained from local producers in Türkiye. Analyses included total soluble solids, hydroxymethylfurfural (HMF), sugars, vitamin C, and essential amino acid content. All values were expressed on a fresh weight basis. The total soluble solids ranged from 55.36% to 80.92%, and total sugar content varied between 40.74 and 44.88 g/100 g. Fructose was the dominant sugar in all samples (15.71–17.64 g/100 g). Vitamin C was the most abundant vitamin, with concentrations ranging from 18.74 to 109.69 mg/kg. Among essential amino acids, arginine was the most prevalent (209.64–483.99 mg/100 g), followed by threonine and methionine. Arginine, important for growth hormone synthesis, may support dietary needs in growing children. PT1 (andiz) had the lowest HMF and the highest vitamin C content, while PT5 (black mulberry) contained the highest levels of all essential amino acids except phenylalanine. Principal Component Analysis (PCA) indicated that PC1 and PC2 accounted for 44.7% and 26.3% of the total variance, respectively, revealing key differentiators among the samples. These findings emphasize the nutritional richness of pekmez and support its promotion as a functional food. The results may also guide local producers in diversifying pekmez production to enhance nutritional value and expand market potential.

## Graphical abstract

This study examines the nutritional profile and functional characteristics of eight traditionally produced pekmez varieties, focusing on essential amino acids, vitamin content, and bioactive compounds. The results highlight the high arginine and vitamin C content, suggesting potential health benefits. Additionally, Principal Component Analysis (PCA) reveals key factors contributing to the variability among pekmez samples.

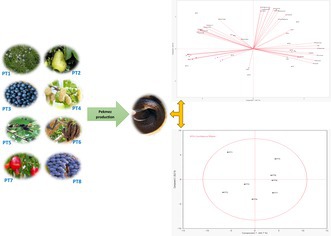

## 1 Introduction

Fruit molasses, known as “Pekmez” in Türkiye, is a traditionally produced and widely consumed food product in Anatolia, a region with a rich culinary heritage. Pekmez is typically produced by boiling and concentrating the juice extracted from various fresh or dried fruits such as grapes, figs, raisins, mulberries, apples, and sugar beets, using open vessels or vacuum systems. Although the production method may vary depending on the type of fruit used, the general process includes pressing, extraction, clarification, filtration, and evaporation steps. The final product usually contains 65%–80% soluble dry matter (Beykaya and Artık 2020).

Pekmez has been produced for centuries in Türkiye and is especially prevalent in regions such as Malatya, Zile, Gaziantep, Nevşehir, Erzincan, and Gümüşhane. However, differences in sensory and chemical characteristics, such as color, taste, and composition, have been reported across different production regions (Cakmakci and Tosun 2010; Türkben et al. 2016).

Due to its high content of glucose, fructose, vitamins, minerals, bioactive compounds, and organic acids (Göğüş et al. 2016), pekmez is considered an important energy source and nutritional component. As a rapidly absorbed sugar source, it is particularly beneficial for individuals such as infants, children, and athletes, and in conditions requiring quick energy supply. Moreover, pekmez is rich in iron and calcium, contributing to bone development and anemia prevention. It also exhibits antioxidant activity and immune‐supporting properties due to its bioactive components (Özcan et al. 2015; Salık et al. 2021; Türkben et al. 2016). When consumed during cold weather, it helps to regulate body temperature and further strengthens the immune system, showing protective effects against various diseases (Kristek et al. 2010; Jain and Venkatasubramanian 2017). Therefore, this traditional food can play a significant role in the diet of various age groups, particularly children and athletes.

Previous studies have mainly focused on pekmez made from commonly consumed fruits like grapes and mulberries (Salık et al. 2021; Sengül et al. 2005; Türkben et al. 2016). However, there is limited research on pekmez derived from alternative fruits such as carob (Tounsi et al. 2020, 2017), pear (Şen et al. 2020), and pomegranate (Altiok et al. 2022). To date, no comprehensive studies have analyzed the chemical composition of aronia ( *Aronia melanocarpa* ) or andiz ( *Juniperus drupacea* ) pekmez. Furthermore, there is a lack of comparative data on essential amino acid, vitamin, hormone, and HMF content across different pekmez types.

Therefore, the aim of this study is to comprehensively evaluate and compare the pH, total soluble solids, sugar profile, amino acid composition, minerals and vitamins, hormones, and HMF (5‐hydroxymethylfurfural) concentration of eight different types of pekmez produced from grape, white mulberry, black mulberry, carob, pomegranate, pear, aronia, and andiz fruits. This study represents the first report of the detailed composition of aronia and andiz pekmez, contributing novel insights to the scientific literature.

## 2 Materials and Methods

### 2.1 Pekmez Samples

*Juniperus drupacea* and *Ceratonia siligua* fruits were harvested from late September to early October in the Mediterranean Region of Türkiye (Alanya) to produce andız and carob pekmez, respectively. Pear pekmez was obtained from Pyrus fruits, which were harvested in the Black Sea Region of Türkiye (Giresun) at the end of August. Aronia pekmez was produced from *Aronia melanocarpa* (chokeberry) fruits, located in the Central Anatolia Region of Türkiye (Konya). These fruits were also harvested in August. *Morus alba* and *Morus nigra* fruits were collected in early July by people who lived in the Eastern Anatolia Province (Erzincan) of Türkiye to produce mulberry and black mulberry pekmez, respectively. *Punica granatum* fruit was used to manufacture pomegranate pekmez in the Eskişehir province of Türkiye in September. Grape pekmez was produced from *Vitis vinifera* fruits, obtained from the Karaman province of Türkiye in September. Traditional methods were used to produce all pekmez types, with small differences for the different fruits. To better monitor the traditional production stages, some of the authors accompanied the manufacturer of all pekmez. While observing the traditional production stages of pekmez, it was noted that the first step of pekmez types production was the selection and washing of the fruits. For each pekmez production, water and fruits were homogenously mixed, heated for about 30 min, and stirred on a firebrand. The mixture is then cooled (approximately 40°C–50°C), pressed, and concentrated for about 2–3 h.

For each type of pekmez, samples from three independent production days were collected and analyzed.

### 2.2 pH, Total Soluble Solid, and HMF Content Determination

pH values and total soluble solid content of pekmez types were determined according to Cemeroğlu (1992). The pekmez samples' 5‐HMF concentrations were ascertained using a modified Zappala et al. (2005) approach. There was no use of heat or acid in the new procedure. Samples were prepared and homogenized in this regard. Each of the Carrez III solutions was added to screw‐capped test tubes in 1.5 mL increments after 10 g of the molasses sample had been diluted to 50 mL with distilled water. After that, a centrifugation process was carried out for 15 min at 5000 rpm. Then, using a 0.22 m syringe filter, the supernatant was transferred to an HPLC system (Agilent 1100, USA) that had a C18 column, 5 μm, and 4.6 mm × 250 mm. 95% was the injection volume, flow rate, and mobile phase. Total soluble solid and HMF content were calculated based on the fresh weight of the pekmez samples.

### 2.3 Sugar Content Determination

Chemicals for sugars (rhamnose, glucose, galactose, xylose, maltose, fructose, and sucrose) in the samples were purchased from Sigma (Steinheim, Germany). Sugar extraction was conducted as described by Nikolidaki et al. (2017). A weighted quantity of mechanically homogenized samples (2 g) was extracted with aqueous ethanol (20 mL, 80% v/v). Sonication and overnight agitation of samples for 2.5 h were applied. For the analysis of sugars in samples, an HPLC system (Agilent Technologies, 1100 series, USA) combined with a refractive index detector (RID, 1260 series) and equipped with an auto‐sampler, an isocratic pump, and a data analysis software was used. Isocratic elution was performed using water/acetonitrile (30:70) on a Purospher star NH2 (250 × 4.6 mm, 5 μm) (MerckMillipore, Darmstadt, Germany) column at a flow rate of 1 mL min^−1^. The injection volume was 10 μL, and RID and oven were maintained at 40°C. Sample determination was performed using calibration standards of HPLC‐grade (Sigma‐Aldrich, Shanghai, China) sugars. Sugars in pekmez types were calculated based on the fresh weight of the pekmez samples.

### 2.4 Vitamin Content Determination

During the analysis, samples were first weighed, and then they were combined with 2.5 mL of an extraction solution, which differed based on the specific analysis: 8% acetic acid for MPA–acetic acid extraction, 0.1% oxalic acid for oxalic acid extraction, and 3% for MPA. This mixture underwent titration with an indophenol solution (comprising 25% DCIP and 21% NaHCO_3_ in water) until a distinct rose‐pink color appeared. For vitamin A analysis, 0.5 g samples were immersed in 20 mL of ethanol and subjected to a 30‐min water bath at 85°C. The cooled solution was then filtered through a separator funnel. Subsequently, heptane (10 mL) was added to the solution, followed by a 5‐min shaking. To allow for layering, 20 mL of a 1.25% sodium sulfate solution was introduced into the tubes, and a 2‐min shaking was conducted. The total tocopherols in the samples were determined through their reaction with cupric ions and complexation with 2,20‐biquinoline (cuproine), following the procedure outlined by Samydurai et al. (2013). The solution was then poured into a conical flask, to which 25 mL of the extraction solution was added. A shaking water bath at 70°C for 40 min was employed to sonicate the solution. Subsequently, after cooling, the samples were filtered with the extraction solution to reach a final volume of 50 mL. The solution for samples underwent further filtration using 0.45 μm filter tips, and 20 μL aliquots of the solution were injected into the HPLC using an autosampler. An analytical reversed‐phase C‐18 column (STR ODS‐M, 150 mm × 4.6 mm I.D., 5 μm, Shimadzu Corporation, Tokyo, Japan) was utilized for the separation of B complex vitamins in the samples. The mobile phase consisted of a mixture of 100‐mM sodium phosphate buffer (pH 2.2) containing 0.8‐mM sodium‐1‐octane sulfonate and acetonitrile at a 9:1 (v/v) ratio at 40°C. The flow rate was maintained at a constant 0.8 mL min^−1^, and a PDA detector was employed with an absorption wavelength of 270 nm. The detection and quantification of B vitamins were performed according to the methodology described by Mozumder et al. (2019). Identifications were performed by spiking samples with pure compounds and comparing retention times (tR) with those of standards. Vitamin A, B1, B2, B6, and C in pekmez types were calculated based on the fresh weight of the pekmez samples.

### 2.5 Mineral Content Determination

Pekmez samples were dried in an oven at 68°C for 48 h. After drying, the samples were ground into a fine powder. The total nitrogen content in the samples was determined using the Kjeldahl method. A Vapodest Rapid Kjeldahl Distillation Unit (Gerhardt, Königswinter, Germany) was employed for the distillation process. The method followed was in accordance with AOAC guidelines. Macroelements, including potassium (K), magnesium (Mg), phosphorus (P), sodium (Na), and calcium (Ca), as well as microelements, such as iron (Fe), zinc (Zn), sulfur (S), chlorine (Cl), copper (Cu), manganese (Mn), and boron (B), were detected using an inductively coupled plasma spectrophotometer (Optima 2100 DV, Perkin‐Elmer, Shelton, CT, USA). The analytical procedure was performed in accordance with the guidelines specified by AOAC (2005). Minerals in pekmez types were calculated based on the fresh weight of the pekmez samples.

### 2.6 Essential Amino Acid Content Determination

Essential amino acid analysis in samples was performed using an Agilent HPLC (HP1100 system, Agilent Technologies Inc.) equipped with a diode array detector (DAD). A Zorbax Eclipse AAA analytical (4.6 mm × 150 mm, 5 μm Agilent Technologies Inc.) column was used for amino acid determination in raisin varieties. An autosampler (G1313A, Agilent Technologies Inc.) was utilized for the inline derivatization by 9‐fluorenylmethyl chloroformate (FMOC) and o‐phthalaldehyde (OPA) immediately prior to injection onto the columns, as described in detail by Henderson et al. (2000). Chromatographic conditions were followed according to the methods described by some authors (Henderson et al. 2000; Lee et al. 2021; Schuster 1988). Briefly, OPA‐derivatized amino acids were recorded at 338 nm, whereas FMOC‐derivatized amino acids were registered at 262 nm. Purchased standards of each individual amino acid (Sigma Chemical Co.) were used for quantification and identification (standard external method). Two internal standards were used: sarcosine for FMOC‐derivatized amino acids and norvaline for OPA‐derivatized amino acids. Individual free amino acid values were expressed as 100 g^−1^ of samples dry weight. Essential amino acids in pekmez types were calculated based on the fresh weight of the pekmez samples.

### 2.7 Hormone Content Determination

Analysis for indole‐3‐acetic acid (IAA) and abscisic acid (ABA) was performed according to the procedure of Kojima et al. (2020). Briefly, sample tissues were homogenized and filtered three times into a solution containing 80% ethanol (1 g fresh weight). After this, 200 pmol of 13C6‐IAA and d6‐ABA were added as internal standards to the solution. The solution was then concentrated using a rotary evaporator, adjusted to a pH of 2.8 with dilute hydrochloric acid, and filtered with a 0.22 μm membrane filter. Partition extraction was performed with diethyl ether, which was also concentrated and filtered with a 0.22 μm membrane filter. The extracts were fractionated with Agilent 1200 Series HPLC (Agilent, CA, USA) equipped with an ultraviolet detector. The HPLC column (Zorbax Eclipse‐AAA C‐18 column, Agilent, CA, USA) was isocratically eluted with a solution of 40% ethanol and 0.1% acetic acid. The eluates correspond to the retention times of IAA and ABA that were collected separately. IAA and ABA fractions were dried under reduced pressure. After fractionation, the obtained fractions were further purified with the same HPLC system. The HPLC column was isocratically eluted, as mentioned above.

Fractions of IAA and ABA were injected, collected, and dried under reduced pressure. Chromatographic conditions to identify and quantify plant hormones are described by Kojima et al. (2020). Analysis of gibberellin (GA3) was performed according to the methodology exposed by Kojima et al. (2020). To a solution containing 80% of ethanol (9 g fresh weight), 200 pmol of d2‐GA3 was added. The solution was then concentrated to 20 mL, adjusted to pH 3.5 with dilute hydrochloric acid, and filtered with a 0.22 μm membrane filter. Partition extraction was performed using ethyl acetate according to the exposure by Kojima et al. (2003). Anhydrous sodium sulfate at 1 g 10 mL^−1^ was added to the ethyl acetate layer for dehydration and allowed to stand overnight. The ethyl acetate layer was decanted, concentrated, dissolved in 1 mL of ethanol, and filtered with a 0.22 μm membrane filter. Extracts from the ethyl acetate layer were fractionated using the HPLC system according to Kojima et al. (2003) and Kojima et al. (2020). In addition, extraction, separation, and purification of GA were performed according to the methodologies exposed by Kojima et al. (2003) and Kojima et al. (2020).

Salicylic acid (SA) was analyzed based on the method described by Kim et al. (2019) with some modifications. Raisins were ground to a fine powder using a mortar and pestle in liquid nitrogen, and 100 mg of the sample was mixed with the extraction solvents. SA was separated and quantified using an Agilent 1200 Series HPLC (Agilent, CA, USA) equipped with a photodiode array detector (Model YL9160). A 5 μL sample was injected into the HPLC system Zorbax Eclipse‐AAA C‐18 column (5 μm, 4.6 × 250 mm, Agilent, CA, USA) that was set to 25°C. The mobile phases were 0.3% phosphoric acid in water (v/v, solvent A) and 100% methanol (solvent B). The flow rate was 0.8 mL min^−1^, and the solvent system was programmed as follows: 0% isocratic of solvent B for 5 min, a subsequent gradient of solvent B from 0% to 100% over 40 min, and maintenance at 100% B for 5 min. Data were acquired and analyzed using the YL‐clarity 4.0 software. SA content was calculated using an external standard. Hormones in pekmez types were calculated based on the fresh weight of the pekmez samples.

### 2.8 Statistical Analysis

Minitab software was used to perform ANOVA analysis to determine whether the effects of pekmez types on the pH values, total soluble solids, sugars, vitamins, minerals, essential amino acids, hormones, and HMF content of the samples were significant (*p* < 0.05). For each pekmez type, samples obtained from three independent production days were analyzed in triplicate.

The Principal Component Analysis (PCA) was conducted with JMP software (JMP Pro 16.0, JMP, Cary, NC, USA) to visualize and interpret the associations between pekmez types and various analytical parameters, including sugars, vitamins, enzymes, minerals, and phenolic compounds.

## 3 Results and Discussion

### 3.1 pH Values, Total Soluble Solids, and HMF (5‐Hydroxymethylfurfural) Content of the Pekmez Samples

Table 1 presents the pH values, total soluble solids, and HMF content of all pekmez types. The pH values ranged from 4.97 to 5.93, with no statistically significant differences observed among the samples (*p* > 0.05). Total soluble solids were found to be between 55.36% and 80.92% Brix, and the PT2 sample showed a significantly lower value compared to the others (*p* < 0.05). Similarly, Oral et al. (2012) reported that molasses is typically produced by concentrating the juices of fruits such as grapes, carob, mulberry, and *Juniperus drupacea* (Andız) to achieve a soluble solid content of 70%–80%. According to Turkish legal regulations (Anonymous 2017b), the total soluble solid content of liquid molasses must be at least 68%. Among the pekmez samples analyzed in this study, only PT2 fell below this legal threshold.

**TABLE 1:** pH values, total soluble solid, and HMF content of the pekmez types.

	Pekmez (fruit molasses) types							
	PT1	PT2	PT3	PT4	PT5	PT6	PT7	PT8
pH	5.19 ± 0.16	5.34 ± 0.18	5.24 ± 0.33	4.97 ± 0.11	5.93 ± 0.43	5.35 ± 0.88	5.46 ± 0.40	5.55 ± 0.32
Total soluble solid (%)	80.28 ± 1.26^a^	55.36 ± 3.98^b^	75.09 ± 4.75^a^	80.92 ± 7.59^a^	76.99 ± 2.17^a^	78.57 ± 1.35^a^	73.36 ± 3.03^a^	79.23 ± 2.71^a^
HMF (mg/kg)	2.28 ± 0.11^g^	1054.66 ± 2.82^b^	18.20 ± 0.21^f^	61.71 ± 0.06^e^	221.30 ± 0.52^c^	3.89 ± 0.03^g^	1238.43 ± 0.69^a^	134.53 ± 1.20^d^

*Note:* Mean ± SE. Within the same line, values with different lowercase superscript letters indicate significant differences (*p* < 0.05). PT1: *Juniperus drupacea* fruit (andiz); PT2: pear; PT3: aronia; PT4: white mulberry; PT5: black mulberry; PT6: carob; PT7: pomegranate; PT8: grape HMF: Hydroxymethylfurfural.

According to Table 1, the highest HMF content (1238.43 mg/kg) was detected in sample PT7. However, the lowest (2.28 mg/kg) was measured in sample PT1.

HMF level of grape pekmez is limited to be 75 mg/kg according to (Anonymous 2017a). The HMF levels of pekmez samples in this study did not exceed the limits for sample PT1, PT3, PT4, and PT6.

While HMF is not generally detected in fresh and unprocessed foods (Askar 1984), its concentration has been reported to increase under conditions such as heat treatment (Fallico et al. 2004) or long‐term storage. Therefore, the amount of HMF is a practical parameter for the quality and freshness of such foods. The formation of HMF during storage conditions is influenced by many factors such as the use of metal containers (White Jr 1979), physicochemical properties (pH, total acidity, mineral content) (OumaáAnam 1995), and moisture due to thermal and/or photo‐chemical stress (Spano et al. 2006).

HMF has been identified in various studies as having potential anti‐carcinogenic (Michail et al. 2007), antioxidant (Wang et al. 2010), anti‐proliferative (Zhao et al. 2013), anti‐sickling (Abdulmalik et al. 2005), anti‐inflammatory (Yamada et al. 2011), antiallergenic (Alizadeh et al. 2017), and anti‐apoptotic properties (Shapla et al. 2018). However, exceeding the recommended dietary limits, 1.6 mg/person/day (Anonymous 2011), of HMF can lead to adverse effects in humans such as carcinogenicity, genotoxicity (Severin et al. 2010), and organotoxicity (Morales and Jiménez‐Pérez 2001).

Although the toxicological significance of HMF is not clear due to inconsistent results on genotoxicity and mutagenicity in in vitro studies, high concentrations of HMF have been found to have cytotoxic effects, causing irritation of the eyes, upper respiratory tract, skin, and mucous membranes (Janzowski et al. 2000).

Our results are in line with those of Erbil (2020) who reported values ranging from 4.10 to 7.00 mg/kg when assessing the HMF content in carob molasses. In addition, Türkben et al. (2016) and Erbil (2020) reported HMF content ranging from 5.93 to 762 mg/kg and 11.7 to 219 mg/kg, respectively, in grape molasses, which are consistent with the findings of the present study. Similarly, the HMF levels observed in mulberry molasses were found to be in agreement with those reported by Karataş and Şengül (2018), Ergun et al. (2019), and Erbil (2020).

According to the legal regulations on pekmez in our country (Anonymous 2017b), it has been reported that the amount of HMF in liquid pekmez should be maximum 75 mg/kg and accordingly, it was determined that the HMF amount of PT2, PT5, PT7, and PT8 samples was above the legal limits.

The reasons why the results obtained in this study differ from those in the literature are thought to be the different conditions such as carbohydrate content of fruits, cooking, and storage conditions. On the other hand, the differences in various parameters in pekmez samples produced by traditional methods can be explained by the possibility of an uncontrolled boiling process during production.

### 3.2 Sugar Content of the Pekmez Samples

The sugar content (sucrose, glucose, fructose, rhamnosis, galactose, xylose, arabinose, and total sugar content) of the pekmez types are given in Table 2. The sugar content determines the taste and consistency of pekmez types, while also providing energy and protective effects. However, it should be kept in mind that high sugar content may have negative effects on health, so the amount of consumption should be considered carefully.

**TABLE 2:** Sugar content of the pekmez types (g/100 g).

Sugar	Pekmez (fruit molasses) types							
	PT1	PT2	PT3	PT4	PT5	PT6	PT7	PT8
Sucrose	13.17 ± 0.18^a^	12.26 ± 1.40^a^	10.64 ± 0.03^b^	10.64 ± 0.04^b^	10.67 ± 0.03^b^	10.66 ± 0.05^b^	10.70 ± 0.04^b^	10.69 ± 0.06^b^
Glucose	8.53 ± 0.24^b^	8.93 ± 1.24^b^	10.50 ± 0.17^a^	10.37 ± 0.25^a^	10.81 ± 0.19^a^	10.67 ± 0.28^a^	11.16 ± 0.21^a^	11.00 ± 0.31^a^
Fructose	15.87 ± 0.69^b^	15.71 ± 1.15^b^	16.94 ± 0.15^ab^	16.92 ± 0.39^ab^	17.27 ± 0.16^ab^	17.25 ± 0.44^ab^	17.64 ± 0.18^a^	17.62 ± 0.49^a^
Rhamnosis	0.28 ± 0.14^b^	0.11 ± 0.04^b^	0.05 ± 0.00^b^	0.37 ± 0.55^b^	1.06 ± 0.11^a^	1.05 ± 0.09^a^	1.18 ± 0.12^a^	1.16 ± 0.07^a^
Galactose	0.02 ± 0.00^b^	0.04 ± 0.04^b^	0.08 ± 0.01^b^	0.28 ± 0.34^b^	0.71 ± 0.04^a^	0.67 ± 0.07^a^	0.79 ± 0.04^a^	0.74 ± 0.08^a^
Xylose	0.02 ± 0.00^b^	0.29 ± 0.47^b^	0.87 ± 0.09^a^	0.86 ± 0.07^a^	0.97 ± 0.10^a^	0.96 ± 0.08^a^	1.08 ± 0.11^a^	1.06 ± 0.09^a^
Arabinose	2.55 ± 0.12^a^	1.87 ± 1.11^ab^	0.62 ± 0.03^c^	0.58 ± 0.06^c^	0.69 ± 0.04^c^	0.65 ± 0.07^c^	0.77 ± 0.04^bc^	0.72 ± 0.07^c^
Total Sugar	40.74 ± 4.87	42.30 ± 0.98	41.66 ± 0.79	43.06 ± 2.70	44.88 ± 0.51	44.79 ± 0.42	44.48 ± 1.27	43.84 ± 0.42

*Note:* Mean ± SE. Within the same line, values with different lowercase superscript letters indicate significant differences (*p* < 0.05). PT1: *Juniperus drupacea* fruit (andiz); PT2: pear; PT3: aronia; PT4: white mulberry; PT5: black mulberry; PT6: carob; PT7: pomegranate; PT8: grape.

The total sugar content in the pekmez samples ranged from 40.74 to 44.88 g/100 g, and the difference between the samples was negligible. The major sugars in all pekmez samples were fructose, sucrose, and glucose, respectively (Table 2). Among the samples, the PT7 had the highest amount (17.64 g/100 g) of fructose, while the lowest amount (15.71 g/100 g) was found in the PT2 (*p* < 0.05). The fructose content of all samples was higher compared to that of glucose and sucrose. Samples were compared in terms of sucrose; it was found that PT1 exhibited the highest sucrose content at 13.17 g/100 g, whereas PT3 and PT4 had the lowest at 10.64 g/100 g (*p* < 0.05). PT7 contained the highest glucose level at 11.16 g/100 g, whereas PT1 had the lowest at 8.53 g/100 g (*p* < 0.05).

According to the Turkish Food Codex Communiqué on Grape Molasses (Anonymous 2017b), the Fructose/Glucose (F/G) ratio of grape molasses should be between 0.9–1.1 and the sucrose ratio should not exceed 1%. It was found that the F/G ratio (1.60) and sucrose amount (10.69 g/100 g) of the grape pekmez samples examined in this study exceeded the legal limits.

In other studies on grape molasses, fructose, glucose, sucrose content, and F/G ratio were reported in the range of 14.17%–34.69%, 26.70%–41.11%, 0.16%–16.14%, and 0.49%–0.94%, respectively (Kaya et al. 2012; Türkben et al. 2016).

According to TS 13717 Carob Molasses Standard (Anonymous 2016), only sucrose content should be between 20%–40% (m/m) regarding the sugar composition of the product. However, in other studies on carob molasses, sucrose, glucose, and fructose content were reported between 22%–44.38%, 7.8%–13.23%, and 10.1%–12.2%, respectively (Pazır and Alper 2016; Simsek and Artık 2002).

According to the TS 12001 Mulberry Molasses Standard, type 1 mulberry molasses may contain a maximum of 14% sucrose, while type 2 may contain up to 17% sucrose (Anonymous 1996). Furthermore, the invert sugar ratio (comprising fructose and glucose) is permitted to range from 45% to 54% for type 1 and from 36% to 45% for type 2. The maximum total sugar content is capped at 66% for type 1% and 60% for type 2.

In many previous studies on mulberry molasses, fructose and glucose have not been analyzed as distinct parameters; rather, the total invert sugar content has been assessed. For instance, Karataş and Şengül (2018) reported that the invert sugar content in their mulberry molasses samples ranged from 31.56% to 55.03%, with sucrose levels varying between 12.03% and 31.34%.

Erbil (2020) determined the fructose, glucose, and sucrose content of traditional production andiz molasses samples in the range of 18.70%–22.49%, 17.32%–25.62%, and 0.62%–18.11%, respectively.

Kalaycıoğlu (2023) analyzed glucose, fructose, and F/G ratio on carob, juniper (andiz), pomegranate, black mulberry, and grape pekmez samples by capillary electrophoresis technique. The glucose content ranged from 22.6 to 35.6 g per 100 g, while fructose levels fluctuate between 20.1 g and 35.8 g per 100 g. The F/G ratios were observed to vary between 0.89 and 1.12.

The differences between the data obtained and the literature results may be due to fruit variety, climate and soil conditions, or production differences, and may be explained by inappropriate storage conditions.

A review of the literature revealed a lack of studies addressing the sugar content of pear and aronia pekmez. Consequently, the current study is anticipated to significantly enhance the existing body of knowledge regarding these specific pekmez types.

Recent studies suggest that molasses may exert functional effects beyond its nutritional value, particularly in relation to metabolic disorders. Due to its content of phenolic compounds, minerals (e.g., magnesium, potassium), B‐group vitamins, and essential amino acids, molasses exhibits notable antioxidant, anti‐diabetic, and anti‐obesity properties (Özkan et al. 2023; Yavaş et al. 2023; El Hosry et al. 2023). These components may contribute to improved glucose metabolism, enhanced insulin sensitivity, and reduced inflammatory responses in adipose tissue. Furthermore, certain bioactive compounds in molasses have been associated with modulation of lipid profiles and oxidative stress markers in individuals with metabolic syndrome (Geremew Kassa et al. 2024). Therefore, although its high sugar content requires cautious consumption, molasses may serve as a supportive functional food ingredient for individuals with diabetes, obesity, or related metabolic conditions.

### 3.3 Vitamin Content of the Pekmez Samples

The vitamin (vitamin A, B1, B2, B6, and C) content of the pekmez types examined in this study is presented in Table 3. Vitamin C, which is the most abundant vitamin for all pekmez types, was found to be highest (109.69 mg/kg) in PT1 and lowest (18.74 mg/kg) in PT4. Vitamin A, B1, B2, and B6 were detected in the range of 1.16–5.60 mg/kg, 0.78–3.11 mg/kg, 0.77–3.17 mg/kg, and 0.13–0.65 mg/kg, respectively. These vitamins also had the highest content in PT1.

**TABLE 3:** Vitamin content of the pekmez types (mg/kg).

Vitamin	Pekmez (fruit molasses) types							
	PT1	PT2	PT3	PT4	PT5	PT6	PT7	PT8
Vitamin A	5.60 ± 0.83^a^	3.89 ± 2.18^ab^	1.19 ± 0.27^c^	1.16 ± 0.32^c^	1.33 ± 0.30^c^	1.29 ± 0.36^c^	1.48 ± 0.33^bc^	1.43 ± 0.40^bc^
Vitamin B1	3.11 ± 0.26^a^	1.99 ± 1.04^b^	0.91 ± 0.10^bc^	0.78 ± 0.09^c^	1.01 ± 0.11^bc^	0.88 ± 0.10^c^	1.12 ± 0.12^bc^	0.96 ± 0.11^bc^
Vitamin B2	3.17 ± 0.22^a^	2.30 ± 1.32^ab^	0.82 ± 0.11^c^	0.77 ± 0.09^c^	0.92 ± 0.12^c^	0.87 ± 0.10^c^	1.03 ± 0.14^bc^	0.97 ± 0.11^bc^
Vitamin B6	0.65 ± 0.06^a^	0.49 ± 0.30^a^	0.15 ± 0.03^b^	0.14 ± 0.01^b^	0.15 ± 0.03^b^	0.13 ± 0.01^b^	0.14 ± 0.02^b^	0.13 ± 0.01^b^
Vitamin C	109.69 ± 6.25^a^	68.52 ± 1.43^b^	31.82 ± 1.81^cd^	18.74 ± 0.39^e^	35.77 ± 2.04^c^	21.07 ± 0.44^e^	40.21 ± 2.29^c^	23.68 ± 0.49^de^

*Note:* Mean ± SE. Within the same line, values with different lowercase superscript letters indicate significant differences (*p* < 0.05). PT1: *Juniperus drupacea* fruit (andiz); PT2: pear; PT3: aronia; PT4: white mulberry; PT5: black mulberry; PT6: carob; PT7: pomegranate; PT8: grape.

Upon reviewing the existing literature on pekmez, it is evident that there is limited data regarding the vitamin content of pekmez produced using traditional methods. In a study conducted on traditional and industrial molasses, the vitamin content of traditional mulberry pekmez was reported as 45, 20, 24, and 78 μg/L for vitamins B2, B5, B9, and C, respectively, while vitamins A, E, B3, and B6 were not detected (Karaca 2009).

Therefore, the results of this study are anticipated to contribute significantly to the scientific literature by providing novel insights into the vitamin composition of pekmez produced using traditional processing methods.

### 3.4 Mineral Content of the Pekmez Types

Table 4 shows the mineral content of pekmez varieties. No significant differences (*p* > 0.05) were found among the pekmez varieties in terms of Mg and Fe content. Na was the most concentrated mineral in all pekmez types, with the lowest level found in PT6 (105.79 mg/kg) and the highest in PT5 (400.13 mg/kg). Following Na, the minerals present in the pekmez varieties in the highest concentrations were Mn (10.90–18.69 mg/kg) and Fe (5.48–9.56 mg/kg), respectively. Other analyzed minerals (N, P, K, Ca, Mg, S, Zn, B, and Cu) were poorly represented in all pekmez types.

**TABLE 4:** Mineral content of the pekmez types (mg/kg).

Mineral matter	Pekmez (fruit molasses) types							
	PT1	PT2	PT3	PT4	PT5	PT6	PT7	PT8
N	1.18 ± 0.33	1.48 ± 0.06	1.26 ± 0.12	1.10 ± 0.06	1.13 ± 0.09	1.19 ± 0.10	1.22 ± 0.11	1.32 ± 0.04
P	0.33 ± 0.06^ab^	0.41 ± 0.03^a^	0.40 ± 0.06^a^	0.34 ± 0.19^ab^	0.14 ± 0.01^b^	0.14 ± 0.00^b^	0.14 ± 0.02^b^	0.16 ± 0.03^b^
K	1.24 ± 0.41^ab^	1.60 ± 0.03^a^	1.37 ± 0.20^ab^	1.04 ± 0.08^b^	0.93 ± 0.10^b^	0.99 ± 0.05^b^	1.05 ± 0.05^b^	1.11 ± 0.14^ab^
Ca	1.17 ± 0.38^ab^	1.48 ± 0.11^a^	1.20 ± 0.08^ab^	0.88 ± 0.26^bc^	0.46 ± 0.05^c^	0.49 ± 0.02^c^	0.51 ± 0.03^c^	0.55 ± 0.04^c^
Mg	0.08 ± 0.04	0.11 ± 0.01	0.08 ± 0.01	0.10 ± 0.05	0.12 ± 0.02	0.12 ± 0.00	0.11 ± 0.02	0.10 ± 0.02
S	0.18 ± 0.05^abc^	0.12 ± 0.04^abc^	0.23 ± 0.06^ab^	0.25 ± 0.12^a^	0.09 ± 0.01^bc^	0.09 ± 0.01^bc^	0.08 ± 0.01^c^	0.08 ± 0.01^c^
Mn	16.19 ± 2.37^ab^	18.69 ± 0.92^a^	15.56 ± 1.09^abc^	15.43 ± 3.34^abc^	10.90 ± 0.78^c^	11.90 ± 1.07^bc^	12.03 ± 0.83^bc^	12.72 ± 2.10^bc^
Fe	7.49 ± 3.46	9.56 ± 1.12	7.80 ± 1.60	6.39 ± 0.62	5.57 ± 0.56	5.77 ± 0.67	5.48 ± 0.15	5.79 ± 0.49
Zn	1.52 ± 0.19^a^	1.80 ± 0.15^a^	1.56 ± 0.27^a^	1.21 ± 0.59^ab^	0.55 ± 0.10^b^	0.59 ± 0.04^b^	0.61 ± 0.16^b^	0.75 ± 0.11^b^
B	1.03 ± 0.08^b^	0.82 ± 0.23^b^	1.31 ± 0.44^ab^	2.88 ± 3.15^ab^	4.77 ± 1.07^a^	4.20 ± 0.62^ab^	3.68 ± 0.17^ab^	3.54 ± 0.88^ab^
Cu	0.03 ± 0.00^c^	0.04 ± 0.00^c^	0.05 ± 0.00^c^	0.04 ± 0.01^c^	0.04 ± 0.00^c^	0.41 ± 0.02^b^	0.58 ± 0.07^a^	0.57 ± 0.05^a^
Na	268.89 ± 8.62^c^	251.08 ± 6.85^c^	314.13 ± 13.90^b^	279.27 ± 9.73^c^	400.13 ± 11.03^a^	105.79 ± 0.43^e^	155.00 ± 0.49^d^	131.45 ± 5.30^de^

*Note:* Mean ± SE. Within the same line, values with different lowercase superscript letters indicate significant differences (*p* < 0.05). PT1: *Juniperus drupacea* fruit (andiz); PT2: pear; PT3: aronia; PT4: white mulberry; PT5: black mulberry; PT6: carob; PT7: pomegranate; PT8: grape.

The pear pekmez studied by Şen et al. (2020) included 515 mg/kg Na, 6594.2 mg/kg K, 5630 mg/kg Ca, and 689 mg/kg Mg; the same minerals were reported as 82.9 mg/kg, 11,979.1 mg/kg, 638.8 mg/kg, and 559.2 mg/kg, respectively, in the study of Öztürk et al. (2023).

While no study on aronia (black chokeberry) pekmez was found in the literature, Kalıčanın et al. (2022) determined approximately 4000 mg/kg K, 500 mg/kg Mg, 1200 mg/kg Ca, and 250 mg/kg Na in a study on basic minerals in aronia berries, juice, and in the dried pomace. Sidor and Gramza‐Michałowska (2019) and Babaoğlu et al. (2022) stated in their studies that aronia fruit is a natural source of antioxidants.

Furthermore, Olcay et al. (2024) investigated the physicochemical properties, nutrient content, and consumer acceptance of sour concentrate and leather products obtained from chokeberry ( *Aronia melanocarpa* ) fruit. Since the total phenolic content of sour concentrate and fruit leather was found to be 2835.52 ± 258.70 mg GAE for 100 g DM and 2043.36 ± 59.60 mg GAE for 100 g DM, respectively, the authors declared that sour concentrate and fruit leather have high antioxidant activity.

The highest mineral concentrations were found in mulberry fruits and pekmez, with calcium levels ranging from 1873.65 to 4437.52 mg/kg, potassium from 10,860 to 15,269 mg/kg, magnesium from 904.48 to 1033.11 mg/kg, sodium from 115.29 to 252.33 mg/kg, phosphorus from 1831 to 2329 mg/kg, and sulfur from 493.35 to 642.25 mg/kg. Ca values for the pekmez samples ranged from 135.76 to 575.84 mg/kg, K from 5.853 to 8.146 g/kg, Mg from 187.96 to 389.86 mg/kg, Na from 20.40 to 43.55 mg/kg, P from 285.10 to 517.72 mg/kg, and S from 121.21 to 303.57 mg/kg. In general, it has been observed that mulberry fruits and pekmez samples include elements that are essential for human metabolism, and K was higher than other minerals (Akbulut and Özcan 2009).

In a study conducted to characterize carob pekmez, K, P, Mg, and Ca content was determined as 1057.3 mg/100 g, 77.8 mg/100 g, 55.6 mg/100 g, and 314.9 mg/100 g, respectively (Tetik et al. 2010).

Some quality parameters of pomegranate pekmez produced by different methods (open pan evaporation, application of activated carbon prior to open pan evaporation, and vacuum evaporation) were investigated, and the Mg, P, Ca, Na, and K content of pekmez samples was reported to be 765.2 mg/kg, 432 mg/kg, 1016 mg/kg, 223 mg/kg, and 8160 mg/kg, respectively, when prepared with open pan evaporation similar to the traditional method.

In a study investigating the mineral content of six different pekmez varieties, namely taflan, pear, black grape, white grape, kiwi, and apple, the Na, K, Mg, and Ca content of black grape and white grape pekmez was reported as 133.3 and 38.9 mg/kg, 9372.3 and 810.1 mg/kg, 558.5 and 450.5 mg/kg, and 961.7 and 440.8 mg/kg, respectively (Öztürk et al. 2023). When the studies on *Juniperus drupacea* (andiz) pekmez were analyzed, the amount of K, Ca, Mg, P, Na, Fe, Cu, Mn, and Zn was reported in the range of 385–18,840, 27,4–1881.8, 11.4–843.8, 1248.2–1445, 35.5–467.6, 2.92–13.60, 1.48–3.73, 0.30–10.71, and 2.94–31.3 mg/kg, respectively (Akbulut et al. 2008; Akinci et al. 2004; Karaca 2009; Özdemir et al. 2004).

### 3.5 Essential Amino Acid Content of the Pekmez Samples

As shown in Table 5, among the pekmez samples, PT5 contained the highest levels of all essential amino acids, with the exception of phenylalanine. In contrast, PT4 was identified as the sample with the lowest essential amino acid content. The concentrations of essential amino acids across the samples were determined as follows: histidine (62.20–110.37 g/100 g), threonine (156.47–282.60 g/100 g), arginine (209.64–483.99 g/100 g), valine (20.55–74.24 g/100 g), tryptophan (11.39–39.10 g/100 g), isoleucine (44.27–96.54 g/100 g), leucine (27.56–79.86 g/100 g), methionine (83.26–238.05 g/100 g), lysine (66.11–123.68 g/100 g), and phenylalanine (6.18–15.18 g/100 g).

**TABLE 5:** Essential amino acid content of the pekmez types (g/100 g).

Amino acid	Pekmez (fruit molasses) types							
	PT1	PT2	PT3	PT4	PT5	PT6	PT7	PT8
Histidine	99.43 ± 5.41^ab^	69.04 ± 5.03^cd^	88.41 ± 5.85^b^	62.20 ± 4.53^d^	110.37 ± 6.00^a^	98.13 ± 6.50^ab^	85.07 ± 6.20^bc^	108.93 ± 7.21^a^
Theonine	261.66 ± 42.43^ab^	168.98 ± 10.93^c^	203.72 ± 29.25^abc^	156.47 ± 10.12^c^	282.60 ± 45.83^a^	220.02 ± 31.59^abc^	197.10 ± 12.75^bc^	237.62 ± 34.11^abc^
Arginine	436.03 ± 46.89^ab^	232.70 ± 21.02^de^	339.03 ± 37.20^bcd^	209.64 ± 18.94^e^	483.99 ± 52.04^a^	376.32 ± 41.30^bc^	286.71 ± 25.90^cde^	417.71 ± 45.84^ab^
Valin	68.74 ± 3.80^a^	22.20 ± 0.94^c^	36.37 ± 2.44^b^	20.55 ± 0.87^c^	74.24 ± 4.09^a^	39.28 ± 2.64^b^	25.89 ± 1.10^c^	42.42 ± 2.85^b^
Tryptophan	36.20 ± 5.30^ab^	12.31 ± 0.25^c^	26.78 ± 3.61^b^	11.39 ± 0.23^c^	39.10 ± 5.72^a^	28.92 ± 3.90^ab^	14.35 ± 0.29^c^	31.23 ± 4.21^ab^
Isoleucine	86.98 ± 10.78^ab^	49.14 ± 3.82^d^	71.17 ± 5.57^bc^	44.27 ± 3.44^d^	96.54 ± 11.97^a^	79.00 ± 6.18^abc^	60.55 ± 4.71^cd^	87.69 ± 6.86^ab^
Leucine	73.95 ± 4.80^a^	29.76 ± 1.42^c^	46.69 ± 2.48^b^	27.56 ± 1.31^c^	79.86 ± 5.18^a^	50.43 ± 2.68^b^	34.71 ± 1.65^c^	54.46 ± 2.90^b^
Lysine	114.52 ± 9.00^a^	71.40 ± 3.54^b^	69.61 ± 7.52^b^	66.11 ± 3.28^b^	123.68 ± 9.72^a^	75.18 ± 8.12^b^	83.28 ± 4.13^b^	81.19 ± 8.77^b^
Methionine	220.42 ± 20.45^ab^	89.92 ± 5.06^d^	161.68 ± 16.04^c^	83.26 ± 4.68^d^	238.05 ± 22.09^a^	174.61 ± 17.32^c^	104.88 ± 5.90^d^	188.58 ± 18.71^bc^
Phenilealanine	12.54 ± 0.96^b^	6.67 ± 0.54^c^	13.01 ± 1.03^ab^	6.18 ± 0.50^c^	13.55 ± 1.03^ab^	14.05 ± 1.11^ab^	7.78 ± 0.63^c^	15.18 ± 1.20^a^

*Note:* Mean ± SE. Within the same line, values with different lowercase superscript letters indicate significant differences (*p* < 0.05). PT1: *Juniperus drupacea* fruit (andiz); PT2: pear; PT3: aronia; PT4: white mulberry; PT5: black mulberry; PT6: carob; PT7: pomegranate; PT8: grape.

The protein content of pekmez is primarily influenced by the type and variety of the fruit used. Proteins form suspensions with substances such as pectin and polyphenols, which cause cloudiness in the syrup and negatively affect the quality of the final product. Therefore, these proteins are removed from the syrup through clarification and filtration processes (Genç 2017). As a result, the protein content in pekmez is lower than that of the fruit used. Furthermore, there are no specific regulations regarding the protein content of pekmez in the Turkish Food Codex or Turkish Standards. It is believed that the data obtained in this study will contribute to the literature on the essential amino acid content, which plays a key role in the bioavailability of protein in different pekmez varieties.

A review of the existing literature on pekmez reveals a notable gap in information regarding the amino acid composition and concentrations of pekmez samples derived from fruits such as *Juniperus drupacea* (andiz), pear, aronia, white mulberry, black mulberry, carob, pomegranate, and grape. To the best of the authors' knowledge, this study represents the first report on the amino acid content of these specific pekmez types. Therefore, the findings of our research contribute significantly to the existing knowledge in this field. On the other hand, in a study conducted on pekmez varieties, the protein amount of traditional production grape, carob, mulberry, and pear pekmez samples were found to be 0.81%, 1.10%, 1.47%, 0.65%, and 1.13%, respectively (Erbil 2020).

In previous studies on pekmez, protein content was reported in the range of 0.63%–2.42% in grape pekmez (Yiğit 2016), 0.33%–1.18% in carob pekmez (Tetik et al. 2010), 0.26%–2.57% in mulberry pekmez (Aksu and Nas 1996; Karataş and Şengül 2018), and 0.61%–0.72% in *Juniperus drupacea* (andiz) pekmez (Akbulut et al. 2008; Akinci et al. 2004).

### 3.6 Hormone Content of the Pekmez Types

Plant hormones, such as auxins, gibberellins, and cytokinins, are typically studied in agronomy and plant sciences. However, these hormones may also have indirect effects on the functional properties and quality of plant‐based food products. This section presents an analysis of the hormone content in various pekmez samples, contributing to a broader understanding of the chemical diversity of pekmez. It should be noted that these compounds do not have direct implications for human nutrition.

Plant hormones are organic compounds that occur naturally in the plant, can be transported from where they are synthesized to other parts of the plant, and can be effective even at very low concentrations. Natural plant hormones and their synthetic counterparts are called Plant Growth Regulators. Synthetic hormones are compounds that are not present in the plant but regulate growth like hormones when applied to the plant (Rademacher 2015). The hormone content of the pekmez samples is given in Table 6.

**TABLE 6:** Hormone content of the pekmez types.

Hormones	Pekmez (fruit molasses) types							
	PT1	PT2	PT3	PT4	PT5	PT6	PT7	PT8
IAA, ng/mg tissue	1.97 ± 0.25^a^	1.76 ± 0.30^ab^	1.43 ± 0.10^ab^	1.63 ± 0.10^ab^	1.72 ± 0.22^ab^	1.35 ± 0.08^ab^	1.33 ± 0.09^b^	1.15 ± 0.41^b^
ABA, ng/g DW	126.46 ± 0.59^d^	76.96 ± 3.20^f^	62.34 ± 8.07^g^	96.46 ± 0.53^e^	86.55 ± 1.61^ef^	580.58 ± 0.80^a^	280.55 ± 1.61^b^	197.64 ± 3.21^c^
GA_3_, ng/g DW	2.28 ± 0.17^f^	4.38 ± 0.10^e^	5.45 ± 0.11^d^	6.82 ± 0.13^c^	10.31 ± 0.49^b^	3.51 ± 0.20^e^	10.71 ± 0.72^b^	16.05 ± 0.44^a^
SA, ng/g DW	0.42 ± 0.01^c^	0.35 ± 0.01^d^	0.24 ± 0.02^e^	0.17 ± 0.01^f^	0.14 ± 0.00^f^	1.38 ± 0.02^a^	0.83 ± 0.04^b^	0.78 ± 0.02^b^
Cytokinin, ng/g DW	0.13 ± 0.01^c^	0.08 ± 0.00^d^	0.05 ± 0.00^e^	0.03 ± 0.00^ef^	0.02 ± 0.00^f^	0.28 ± 0.02^a^	0.18 ± 0.01^b^	0.19 ± 0.00^b^
Zeatin, ng/g DW	1.42 ± 0.07^c^	1.03 ± 0.07^d^	0.79 ± 0.01^e^	0.50 ± 0.03^f^	0.42 ± 0.03^f^	2.82 ± 0.14^a^	1.71 ± 0.14^b^	1.78 ± 0.09^b^
Jasmonic acid, ng/g DW	0.41 ± 0.04^c^	0.39 ± 0.03^c^	0.25 ± 0.02^d^	0.21 ± 0.01^de^	0.13 ± 0.02^e^	1.07 ± 0.05^a^	0.85 ± 0.03^b^	0.93 ± 0.04^b^

*Note:* Mean ± SE. Within the same line, values with different lowercase superscript letters indicate significant differences (*p* < 0.05). PT1: *Juniperus drupacea* fruit (andiz); PT2: pear; PT3: aronia; PT4: white mulberry; PT5: black mulberry; PT6: carob; PT7: pomegranate; PT8: grape.

Abbreviations: ABA, abscisic acid; GA, gibberellic acid; IAA, indole‐3‐acetic acid; SA, salicylic acid.

IAA is the only hormone that can be synthesized naturally in plants and belongs to the first discovered group of growth regulators, the auxins (Duca et al. 2014). In pekmez samples, this hormone was determined to be in the range of 1.15–1.97 ng/mg tissue, with the highest in PT1 and the lowest in PT8 samples.

Giberellins, like auxins, are growth and development‐promoting hormones at low doses. Today, at least 126 types of giberellins are known, but the most widely used and commercially important one is gibberellic acid (GA_3_). The most prominent effect of giberellins is to increase cell elongation. In practice, GA_3_ is mostly used to thin the clusters and increase grain size in table and dried grapes (Ghosh and Halder 2018). In line with the literature data, the highest (16.05 ng/g DW) GA_3_ value was determined in grape pekmez samples (PT8) while the lowest (2.28 ng/g DW) GA_3_ value was determined in PT1 samples in this study.

Cytokinins are hormones that initiate cell division, as the name suggests (cytokinensis = cell division). The first plant‐derived cytokinin was zeatin, isolated from corn seeds. Zeatin is a naturally synthesized cytokinin (Algül et al. 2016; Wybouw and de Rybel 2019).

The amounts of cytokinin and zeatin in pekmez varieties ranged from 0.02 to 0.28 ng/g DW and 0.42 to 2.82 ng/g DW, respectively. The highest concentrations of both hormones were found in the PT6 sample, while the lowest concentrations were found in the PT5 sample.

The jasmine ( *Jasminum grandiflorum* ) plant is the original source of jasmonic acid (JA), one of the jasmonates. Flowers, leaves, roots, and immature fruits all produce jasmonates.

Plant resistance mechanisms are particularly strengthened by jasmonates, which also make plants more resilient to pests and diseases. Jasmonates have been shown to facilitate fruit ripening by promoting adventitious root formation, abscission, stomata closure, protein synthesis, resting needs, latent seed germination, and ethylene synthesis (Ghorbel et al. 2021). The amounts of jasmonic acid in pekmez varieties ranged from 0.13 to 1.07 ng/g DW. The highest concentrations of this hormone were found in the PT6 sample, while the lowest concentrations were found in the PT5 sample.

SA gets its name from the willow (Salix) tree, whose leaves and bark have been known for centuries to alleviate pain and fever. SA is present in all parts of the plant and is transported via the phloem from the site of external application to different organs. Numerous studies have shown that SA inhibits ethylene synthesis in apples, enhances grain yield in beans, promotes rooting, and accelerates photosynthesis (Hayat et al. 2007). Some of the most notable applications of SA involve enhancing resistance to harsh environmental conditions, including drought, salinity, extreme temperatures (both high and low), heavy metal exposure, and frost stress (Baktır 2010; Hassoon and Abduljabbar 2019). The amount of SA in pekmez varieties ranged from 0.14 to 1.38 ng/g DW. Among the samples, the PT6 had the highest hormone concentrations, in contrast to the PT5, which had the lowest.

ABA is a natural inhibitor of growth‐promoting hormones such as auxins, gibberellins, and cytokinins. While ABA is found in all plant organs, it is mainly produced in the mesophyll cells of leaves and is most concentrated in green leaves. During stress conditions, the synthesis of ABA increases, and it is quickly transported to other parts of the plant through the petiole and stem tissues. It has been noted that ABA is not commonly used in practice due to the high cost of its synthetic production and its instability under UV light (Baktır 2010). The PT6 sample exhibited the highest (580.58 ng/g DW) concentrations of this hormone, whereas the PT3 sample had the lowest (62.34 ng/g DW) concentrations.

Since no data regarding the hormone content of pekmez types could be found in the existing literature, this parameter could not be discussed in the present study. Nevertheless, the findings presented here are expected to make a valuable contribution to the current body of knowledge in this area.

### 3.7 Principal Component Analysis (PCA) of the Pekmez Samples

**FIGURE 1:**
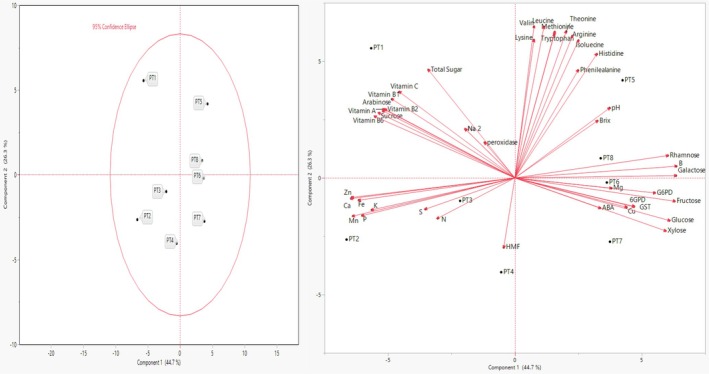
PCA biplot analysis of eight different pekmez samples based on their chemical and nutritional properties.

The PCA results are given in Table 7, and Figure 1 illustrates the relationship between the nutritional and chemical parameters of eight different pekmez samples (PT1 to PT8). The first and second principal components (PC1 and PC2) explain 44.7% and 26.3% of the total variance, respectively, accounting for a cumulative 70.98% of the overall variability.

**TABLE 7:** PCA results of different pekmez samples based on nutritional parameters.

Parameters	PC1	PC2
Eigenvalue	19.68	11.56
Variability (%)	44.72	26.26
Cumulative (%)	44.72	70.98
Correlations		
Vitamin C	−0.155	0.163
G6PD	0.187	−0.028
6GPD	0.159	−0.055
GST	0.159	−0.054
Peroxidase	−0.040	0.067
Na	−0.066	0.092
ABA	0.114	−0.057
N	−0.103	−0.077
P	−0.204	−0.071
K	−0.192	−0.061
Ca	−0.219	−0.039
Mg	0.130	−0.020
S	−0.120	−0.059
Mn	−0.217	−0.072
Fe	−0.209	−0.042
Zn	−0.218	−0.037
B	0.216	0.022
Cu	0.149	−0.056
Na	−0.066	0.092
Sucrose	−0.182	0.123
Glucose	0.207	−0.081
Fructose	0.214	−0.044
Rhamnose	0.205	0.043
Galactose	0.216	0.004
Xylose	0.202	−0.100
Arabinose	−0.176	0.127
Total Sugar	−0.116	0.205
Vitamin A	−0.176	0.131
Vitamin B1	−0.165	0.150
Vitamin B2	−0.174	0.130
Vitamin B6	−0.187	0.117
Histidine	0.110	0.235
Theonine	0.069	0.278
Arginine	0.077	0.270
Valin	0.026	0.287
Tryptophan	0.053	0.273
İsoluecine	0.085	0.261
Leucine	0.039	0.287
Lysine	0.026	0.262
Methionine	0.053	0.277
Phenilealanine	0.085	0.204
pH	0.127	0.133
Brix	0.110	0.108
HMF	−0.015	−0.132

This analysis revealed a clear separation of the samples based on their nutritional and chemical composition. Notably, sample PT1 was distinctly positioned in the upper‐left quadrant, indicating strong negative scores on PC1 and positive scores on PC2. This placement highlights its unique composition compared to the other samples. According to the PCA loadings (Table 7), PT1's separation is primarily influenced by higher levels of parameters that are negatively correlated with PC1 and positively correlated with PC2. These include Vitamin C, Vitamin A, B‐group vitamins (B1, B2, B6), and several minerals such as calcium (Ca), iron (Fe), manganese (Mn), zinc (Zn), and phosphorus (P). The strong presence of these components likely contributes to PT1's distinct positioning on the PCA plot.

In contrast, variables such as fructose, glucose, galactose, rhamnose, and boron (B), which show strong positive correlations with PC1, appear to influence the positioning of other samples toward the right side of the plot. This suggests a compositional divergence between PT1 and the rest, particularly in terms of sugar profile and certain mineral content.

Overall, PCA provided a comprehensive overview of the variation in the nutritional and chemical characteristics of pekmez samples, and clearly demonstrated that PT1 differs significantly from the others due to its specific vitamin and mineral composition. These findings can guide future research and applications related to the targeted nutritional use of different pekmez types.

## 4 Conclusion

In this study, eight different pekmez varieties ( *Juniperus drupacea* fruit (andiz), pear, aronia, white mulberry, black mulberry, carob, pomegranate, and grape) obtained from different parts of Türkiye and produced by traditional methods were analyzed in terms of various parameters such as pH, HMF, total soluble solids, sugars, minerals, vitamins, essential amino acids, and hormone content. One of the most striking parts of the study is that the highest HMF content is found in pomegranate pekmez. Also, while black mulberry pekmez contains high amounts of B and Na minerals, another remarkable result is that the highest amino acid contents, except phenylalanine, were detected in black mulberry pekmez. The PCA results effectively highlighted the key parameters driving variability among pekmez samples, offering valuable insights into their distinct chemical and nutritional compositions. When the results obtained are examined, it is seen that there are some differences between the literature data, but it is thought that these differences are due to variations such as fruit variety, fruit ripeness, and pekmez production methods. Consequently, these findings could underscore the crucial properties of pekmez types, providing consumers with valuable data to consume nutrient‐enriched products. Our results also highlight that the local producers should diversify their pekmez production from different fruits, thus increasing both its nutritional value and market potential.

## Author Contributions

**Nazik Meziyet Dilek:** formal analysis (equal), writing – original draft (equal), writing – review and editing (equal). **Ali Samet Babaoğlu:** formal analysis (equal), investigation (equal), writing – review and editing (equal). **Kubra Unal:** formal analysis (equal), writing – review and editing (equal). **Metin Turan:** formal analysis (equal). **Lütfi Pırlak:** writing – review and editing (equal). **Mustafa Karakaya:** writing – review and editing (equal).

## Ethics Statement

This manuscript does not contain any studies with human participants or animals performed by any of the authors.

## Conflicts of Interest

The authors declare no conflicts of interest.

## Data Availability

The data presented in this study are available on request from the corresponding author. The data are not publicly available.
